# Physical Activity and Sedentary Behaviour in the MEDIET4ALL Study: Associations with Mediterranean Lifestyle, Sleep, and Psychosocial Well-Being, with Mediation Analyses

**DOI:** 10.3390/sports14050186

**Published:** 2026-05-06

**Authors:** Achraf Ammar, Atef Salem, Khaled Trabelsi, Martha Montalvan, Bassem Bouaziz, Mohamed Ali Boujelbane, Mohamed Kerkeni, Liwa Masmoudi, Hadeel Ali Ghazzawi, Adam Tawfiq Amawi, Bekir Erhan Orhan, Raynier Zambrano-Villacres, Juliane Heydenreich, Christiana Schallhorn, Tarak Driss, Evelyn Frias-Toral, Piotr Zmijewski, Haitham Jahrami, Waqar Husain, Hamdi Chtourou, Wolfgang I. Schöllhorn

**Affiliations:** 1 Department of Training and Movement Science, Institute of Sport Science, Johannes Gutenberg-University Mainz, 55122 Mainz, Germany; asalem@uni-mainz.de (A.S.); schoellw@uni-mainz.de (W.I.S.); 2 Research Laboratory, Molecular Bases of Human Pathology, LR19ES13, Faculty of Medicine of Sfax, University of Sfax, Sfax 3000, Tunisia; 3 Interdisciplinary Laboratory in Neurosciences, Physiology, and Psychology: Physical Activity, Health, and Learning (LINP2), UFR STAPS, Paris Nanterre University, 92000 Nanterre, France; tdriss@parisnanterre.fr; 4 Department of Nutrition and Food Technology, School of Agriculture, The University of Jordan, Amman 11942, Jordan; h.ghazzawi@ju.edu.jo; 5 High Institute of Sport and Physical Education of Sfax, University of Sfax, Sfax 3000, Tunisia; mboujelb@uni-mainz.de (M.A.B.); mohamed.kerkeni@isseps.usf.tn (M.K.); hamdi.chtourou@isseps.usf.tn (H.C.); 6 Research Laboratory: Education, Motricity, Sport and Health, EM2S, LR19JS01, High Institute of Sport and Physical Education of Sfax, University of Sfax, Sfax 3000, Tunisia; trabelsikhaled@gmail.com (K.T.); liwa.masmoudi@isseps.usf.tn (L.M.); 7 Department of Movement Sciences and Sports Training, School of Sports Science, The University of Jordan, Amman 11942, Jordan; a.amawi@ju.edu.jo; 8 School of Medicine, Universidad Católica de Santiago de Guayaquil, Av. Pdte. Carlos Julio Arosemena Tola, Guayaquil 090615, Ecuador; mmontalvanmd53@gmail.com; 9 Multimedia InfoRmation Systems and Advanced Computing Laboratory (MIRACL), University of Sfax, Sfax 3000, Tunisia; bassem.bouaziz@isims.usf.tn; 10 Higher Institute of Computer Science and Multimedia of Sfax (ISIMS), University of Sfax, Sfax 3000, Tunisia; 11 Faculty of Sports Sciences, Istanbul Aydın University, Istanbul 34295, Türkiye; bekirerhanorhan@aydin.edu.tr; 12 Facultad de Ciencias de la Salud y Desarrollo Humano, Universidad ECOTEC, Km. 13.5 Samborondón, Samborondón 092302, Ecuador; razambrano@ecotec.edu.ec; 13 Department of Experimental Sports Nutrition, Faculty of Sports Sciences, Leipzig University, 04109 Leipzig, Germany; juliane.heydenreich@uni-leipzig.de; 14 Department of Sports Economics, Sociology and History, Institute of Sport Science, Johannes Gutenberg-University Mainz, 55122 Mainz, Germany; christiana.schallhorn@uni-mainz.de; 15 Escuela de Medicina, Universidad Espíritu Santo, Samborondón 092301, Ecuador; evelynft@gmail.com; 16 Division of Research, Texas State University, 601 University Drive, San Marcos, TX 78666, USA; 17 Department of Biomedical Sciences, Józef Piłsudski University of Physical Education in Warsaw, 00-968 Warsaw, Poland; piotr.zmijewski@insp.waw.pl; 18 Government Hospitals, Manama 323, Bahrain; hjahrami@health.gov.bh; 19 Department of Psychiatry, College of Medicine and Health Sciences, Arabian Gulf University, Manama 329, Bahrain; 20 Department of Humanities, COMSATS University Islamabad, Islamabad 45550, Pakistan; drsukoon@gmail.com; 21 Research Unit, Physical Activity, Sport, and Health, UR18JS01, National Observatory of Sport, Tunis 1003, Tunisia

**Keywords:** sedentary time, mediterranean diet, multinational survey, behavioural epidemiology, social participation, sleep duration, mediation analysis, active lifestyle

## Abstract

**Background/Objectives**: Physical activity and sedentary behaviour represent related yet distinct movement behaviours with potentially different behavioural, psychosocial, and lifestyle correlates. However, multinational evidence examining these behaviours within the Mediterranean lifestyle framework remains limited. This study investigated correlates of physical activity and sedentary behaviour among adults from Mediterranean and neighbouring countries participating in the MEDIET4ALL survey. **Methods**: Data were collected from 4010 adults (37.2 ± 15.4 years; 59.5% female) across 10 Mediterranean and neighbouring countries using a standardized multilingual e-survey. Physical activity was assessed using the short International Physical Activity Questionnaire (IPAQ-SF; MET-min/week), and sedentary behaviour was assessed by daily sitting time. Hierarchical multiple linear regression analyses were conducted separately for physical activity and sedentary behaviour. Exploratory bootstrapped mediation analyses examined whether life satisfaction (SLSQ) or social participation (SSPQ) mediated associations between MEDLIFE dietary dimensions and sitting time. **Results**: Higher physical activity was associated with more rural living environments, lower body mass index, more favourable smoking status, higher alcohol consumption, stronger adherence to Mediterranean dietary habits, longer sleep latency, higher stress, and greater social participation (β ≈ 0.05–0.11), whereas female sex, longer sleep duration, and higher anxiety were associated with lower physical activity (β = −0.04 to −0.23); the positive association with alcohol consumption should be interpreted cautiously in light of potential residual confounding. By contrast, sedentary behaviour was positively associated with higher education, higher body mass index, and more favourable smoking-status (β ≈ 0.04–0.09) and inversely associated with better self-reported health status, Mediterranean dietary consumption patterns, life satisfaction, and social participation (β = −0.04 to −0.07). Mediation analyses showed significant but small-magnitude indirect effects for the pathways linking MEDLIFE dietary consumption patterns and MEDLIFE dietary habits with sitting time through social participation (indirect β = −0.0032 and −0.0045, respectively), which should be interpreted with caution, whereas no significant indirect effects were observed through life satisfaction. **Conclusions**: Physical activity and sedentary behaviour are associated with different, though partially overlapping, lifestyle and psychosocial correlates within the MEDIET4ALL framework. Social participation may represent a modest behavioural pathway linking Mediterranean dietary dimensions with lower sitting time. Given the cross-sectional design, these findings should be interpreted as associative rather than causal, but they nonetheless reinforce the importance of integrated and context-sensitive lifestyle promotion strategies.

## 1. Introduction

Physical inactivity and prolonged sedentary behaviour are major contributors to the global public health burden [1,2]. Insufficient physical activity is strongly associated with increased risks of cardiovascular disease, type 2 diabetes, several types of cancer, and premature mortality and contributes substantially to the global burden of non-communicable diseases [1,3,4]. Estimates from the Global Burden of Disease studies indicate that low physical activity contributes to millions of deaths and disability-adjusted life years worldwide [2], highlighting the urgent need for effective strategies to promote active lifestyles [1,2]. At the same time, sedentary behaviour, defined as any waking behaviour characterized by energy expenditure ≤1.5 metabolic equivalents while in a sitting, reclining, or lying posture [5], has been identified as an independent risk factor for adverse health outcomes even among individuals who meet recommended physical activity guidelines [6,7,8,9]. These concerns are further compounded by broader lifestyle trends, including increasing consumption of ultra-processed foods, declining adherence to traditional dietary patterns, and rising levels of social isolation and psychological stress, all of which may cluster with sedentary behaviour and thereby exacerbate health risks through poorer diet quality, reduced social engagement, and a more adverse metabolic profile [10,11,12,13]. Conversely, regular engagement in moderate-to-vigorous physical activity has consistently been linked to improved cardiometabolic health, enhanced functional capacity, better mental health, and improved overall quality of life [14,15,16]. Despite these well-documented benefits, adherence to recommended physical activity guidelines remains suboptimal worldwide, with approximately one quarter to one third of adults failing to meet minimum recommended activity levels and many individuals spending substantial portions of their daily time in sedentary behaviours [3,17].

Beyond its well-established physical and mental health benefits [14,15,18], physical activity is increasingly recognized as a key behavioural determinant within broader lifestyle patterns influencing health and well-being, particularly within integrated frameworks that consider multiple lifestyle behaviours such as diet, sleep, and sedentary habits [19,20,21]. The Mediterranean lifestyle has been proposed as a multidimensional behavioural model characterized not only by adherence to the Mediterranean diet but also by regular physical activity, adequate sleep, social engagement, and balanced daily routines [12,20,22]. Within this framework, physical activity represents a central behavioural component that historically reflects traditional Mediterranean living patterns involving active transportation, occupational activity, and social participation [12]. However, modernization, urbanization, and changes in occupational and leisure patterns have progressively reduced habitual physical activity levels and increased sedentary behaviour in many populations, including those in Mediterranean regions [3,17,23]. Understanding how physical activity and sedentary behaviour interact with other lifestyle dimensions is therefore essential for developing integrated health promotion strategies [24,25].

Accumulating evidence suggests that physical activity behaviour is shaped by a complex interplay of sociodemographic, behavioural, and psychosocial factors. Previous studies have reported consistent associations between physical activity levels and characteristics such as age, sex, educational attainment, and socioeconomic status [26]. In addition, behavioural factors, including sleep patterns, sedentary habits, and dietary quality, have been shown to be closely associated with physical activity engagement within broader 24 h and lifestyle behaviour frameworks [27,28,29]. Recent evidence highlights the interrelationships between sleep, dietary behaviour, and broader lifestyle patterns, with studies showing that eating behaviours and dietary patterns are associated with sleep quality and sleep health and that these interrelated behaviours may jointly relate to health outcomes such as adiposity [30,31,32]. Psychosocial determinants such as mental well-being, social support, and social participation may also contribute to shaping movement behaviours across the lifespan [33,34,35]. Evidence from Mediterranean populations also supports the interconnected nature of lifestyle behaviours. For example, recent cross-national research conducted among children and adolescents from five Mediterranean countries demonstrated that dietary habits and broader lifestyle patterns are closely associated with physical activity levels, highlighting the interrelated roles of diet, movement behaviours, and lifestyle environments in shaping youth health [36]. However, despite these advances, important knowledge gaps remain, particularly in adult multinational populations where multiple lifestyle domains are examined simultaneously. Much of the existing literature has examined individual lifestyle behaviours in isolation, and studies that simultaneously investigate multiple lifestyle domains across countries remain relatively limited. Moreover, many of the available multinational investigations have focused primarily on children and adolescent populations (e.g., Rosi et al., [36]), leaving the interplay between physical activity, sedentary behaviour, and other lifestyle factors insufficiently explored in adult populations.

The MEDIET4ALL project provides a valuable framework to further explore behavioural determinants of health within diverse Mediterranean and neighbouring populations [37]. The project relies on a standardized international electronic survey designed to assess adherence to the Mediterranean lifestyle together with a wide range of behavioural, psychosocial, and sociodemographic characteristics [19,38]. Previous determinant analyses based on the MEDIET4ALL dataset have revealed that both Mediterranean lifestyle adherence and self-reported health status are shaped by a multidimensional set of demographic, behavioural, and psychosocial factors rather than by dietary behaviours alone [10,19]. These findings highlight the interconnected nature of lifestyle behaviours in shaping health outcomes; however, physical activity and sedentary behaviour have not yet been examined as primary outcome variables within the MEDIET4ALL analytical framework.

Understanding how movement behaviours relate to Mediterranean lifestyle adherence and other lifestyle dimensions, including sleep patterns, psychosocial characteristics, and perceived barriers, may provide important insights into the behavioural factors shaping active and sedentary lifestyles. Such insights could contribute to the development of integrated lifestyle-based strategies aimed at promoting physical activity and reducing sedentary behaviour in adult populations. Therefore, the present study aimed to investigate the correlates of physical activity and sedentary behaviour as key behavioural outcomes within the MEDIET4ALL international survey. A specific focus was placed on their associations with Mediterranean lifestyle adherence and selected behavioural correlates including sleep characteristics, psychological well-being, social participation, and technology use behaviours, as well as on the potential mediating roles of life satisfaction and social participation.

## 2. Materials and Methods

### 2.1. Study Design and Participants

This study draws on data collected within the MEDIET4ALL project, a multinational cross-sectional investigation designed to evaluate adherence to the Mediterranean lifestyle and its behavioural, psychosocial, and sociodemographic correlates across Mediterranean and neighbouring populations. Data from 4010 responders were obtained through a standardized electronic survey administered in ten participating countries such as Germany (n = 616), France (n = 533), Italy (n = 711), Spain (n = 278), Luxembourg (n = 118), Tunisia (n = 170), Algeria (n = 146), Morocco (n = 160), Türkiye (n = 596), and Jordan (n = 682). The use of an online survey enabled the collection of harmonized information across different sociocultural contexts while facilitating large-scale participation. The questionnaire was distributed through consortium and partner networks as well as institutional websites, mailing lists, and social media platforms.

The methodological framework and survey development procedures of the MEDIET4ALL project have been described in detail elsewhere [10,19,38,39,40,41]. In brief, the survey targeted adults aged 18 years or older living in participating countries. The survey was implemented using the secure GDPR-compliant SoSci Survey platform hosted at Johannes Gutenberg University Mainz and was available in multiple languages to maximize participation across countries (English, German, French, Italian, Spanish, Arabic, and Turkish). When validated translations were not available for specific survey’s instruments, translation and back-translation procedures were conducted following established cross-cultural adaptation guidelines [42]. Reliability testing indicated excellent test–retest consistency across language versions, with correlation coefficients ranging between 0.81 and 0.94. Participation was voluntary and anonymous, and all respondents provided electronic informed consent before accessing the questionnaire.

The final analytical sample consisted of 4010 respondents. Prior to statistical analysis, the dataset underwent systematic screening procedures to ensure data quality. These procedures included verification of complete questionnaire submission, removal of incomplete entries, logical consistency checks across related variables, identification of potential duplicate responses based on IP address and response timestamps, and the exclusion of implausible values. Only responses meeting predefined completeness and consistency criteria were retained for analysis [10,19,38,39,40,41].

The study protocol complied with the Declaration of Helsinki and received approval from the Ethics Committee of the Faculty of Medicine, University of Sfax (Approval Code: 058/24). Additional details regarding recruitment procedures, survey administration, and data privacy measures have been reported in previous MEDIET4ALL publications [10,38,39,40,41].

### 2.2. Survey Instruments and Measured Variables

The MEDIET4ALL questionnaire was developed to assess multiple components of the Mediterranean lifestyle together with relevant sociodemographic characteristics, health behaviours, and psychosocial variables.

#### 2.2.1. Sociodemographic and Lifestyle Characteristics

Participants reported demographic information including age, sex, country of residence, education level, employment status, marital status, and living environment. Lifestyle characteristics included smoking status, alcohol consumption, and self-reported health status. Anthropometric data (body weight and height) were self-reported and used to calculate body mass index (BMI) [10,19,38,39]

#### 2.2.2. Physical Activity and Sedentary Behaviour

Physical activity was assessed using the short form of the International Physical Activity Questionnaire (IPAQ-SF), which estimates weekly physical activity energy expenditure expressed in metabolic equivalent task minutes per week (MET-min/week) across walking, moderate-intensity, and vigorous-intensity activities. Participants also reported, using a single self-reported item, their average daily sitting time, which was used as an indicator of sedentary behaviour. The IPAQ-SF has been widely applied in international surveillance studies and has demonstrated acceptable reliability and validity for assessing population-level physical activity patterns [43]. Nevertheless, IPAQ-SF is known to overestimate physical activity levels compared with objective measures such as accelerometry [44].

#### 2.2.3. Mediterranean Lifestyle Adherence

Adherence to the Mediterranean lifestyle was assessed using the Mediterranean Lifestyle Index (MEDLIFE), a validated instrument developed to capture key behavioural characteristics of the traditional Mediterranean lifestyle [45]. The MEDLIFE index evaluates three main domains: (i) Mediterranean dietary consumption patterns, (ii) Mediterranean dietary habits, and (iii) lifestyle behaviours including physical activity, rest, social interaction, and conviviality. Each item is scored according to the standardized MEDLIFE scoring procedure, with higher scores reflecting greater adherence to Mediterranean lifestyle practices [19,45].

In the present study, the two dietary domains of the MEDLIFE index, Mediterranean dietary consumption patterns and Mediterranean dietary habits, were analyzed separately. The third domain, which includes items related to physical activity and rest, was not included in the present analyses in order to avoid conceptual overlap between the exposure variables and the study outcomes, namely physical activity and sedentary behaviour, and prevent collinearity between predictor and outcome variables. This approach allows the examination of dietary-related Mediterranean lifestyle components while preserving the independence of the movement behaviour outcomes.

#### 2.2.4. Sleep Characteristics and Insomnia

Sleep-related information was collected using selected indicators adapted from established sleep assessment tools, including items derived from the Pittsburgh Sleep Quality Index (PSQI) together with the Insomnia Severity Index (ISI). Four principal sleep dimensions were evaluated [10,38,39,40,41]. Sleep efficiency was calculated as the ratio of total sleep time to time spent in bed and categorized using a threshold of >85% versus ≤85%. Sleep latency was classified according to time required to fall asleep (<20 min vs. ≥20 min). Subjective sleep quality was assessed on a four-point scale ranging from “very good” to “very bad,” while sleep duration categories were defined according to age-specific sleep recommendations.

Insomnia symptoms were assessed using the Insomnia Severity Index (ISI), a validated seven-item instrument measuring difficulties with sleep initiation, sleep maintenance, early awakenings, and the perceived impact of sleep problems on daily functioning [46]. Each item is scored from 0 to 4, yielding a total score between 0 and 28, with higher scores indicating greater insomnia severity.

#### 2.2.5. Social Participation and Technology Use

Social engagement was assessed using the Short Social Participation Questionnaire (SSPQ), which evaluates participation in social activities and interpersonal interactions. The questionnaire comprises 14 items, including ten Likert-scale items and four dichotomous questions. Total scores range from 14 to 70, with higher values reflecting greater social participation.

Technology use was measured using the Short Technology-Use Questionnaire (STuQL), which assesses the frequency of technology use in relation to lifestyle behaviours such as social interaction, dietary practices, and physical activity. The STuQL contains three items rated on a five-point scale ranging from “never” to “all the time,” resulting in scores from 3 to 15, with higher scores indicating more frequent technology use.

Both instruments have previously been applied in previous MEDIET4ALL analyses [10,19,38,39,40,41].

### 2.3. Statistical Analysis

All statistical analyses were performed using SPSS (version 29; Chicago, IL, USA). Descriptive statistics were calculated to summarize participant characteristics and the distribution of study variables. To examine factors associated with movement behaviours, separate hierarchical multiple linear regression analyses were conducted for physical activity and sedentary behaviour. Physical activity was operationalized as weekly energy expenditure derived from the IPAQ-SF (MET-min/week), while sedentary behaviour was assessed using daily sitting time. Because the distribution of physical activity values was positively skewed, MET-min/week values were log-transformed (log(MET-min/week + 1)) prior to regression analyses. Daily sitting time was analyzed as a continuous outcome.

The regression analyses followed a hierarchical block modelling strategy in which predictors were entered sequentially in conceptually defined groups. This approach enabled the progressive adjustment of behavioural and psychosocial predictors for potential confounding by sociodemographic and health-related characteristics while allowing the evaluation of the incremental contribution of each predictor block to the explained variance in movement behaviour outcomes.

Model 1 included sociodemographic variables (Block 1), namely age, sex, country or region, education level, employment status, marital status, and living environment. Model 2 extended this model by additionally including health-related variables (Block 2), specifically body mass index (BMI), smoking status, alcohol consumption, and self-reported health status. Model 3 incorporated Mediterranean lifestyle-related dietary behaviours (Block 3), operationalized using the first and second dietary domains of the MEDLIFE index: Mediterranean dietary consumption patterns and Mediterranean dietary habits scores. Model 4 included sleep-related variables (Block 4), comprising sleep duration, sleep latency, sleep efficiency, subjective sleep quality, and insomnia severity. Model 5 added psychological variables (Block 5), including life satisfaction and psychological distress indicators. Model 6 incorporated social participation and technology use (Block 6).

This hierarchical modelling strategy allowed the examination of associations between Mediterranean lifestyle-related dietary behaviours and movement outcomes after sequential adjustment for sociodemographic and health-related factors. A final parsimonious model (Model 7) was constructed by retaining predictors that remained statistically significant (*p* < 0.05) in the fully adjusted model (model 6).

In addition, exploratory mediation analyses were conducted to examine selected indirect associations between Mediterranean dietary lifestyle components, psychosocial factors, and sedentary behaviour within the MEDIET4ALL sample. Informed by the regression analyses, mediation models were used to test whether life satisfaction or social participation mediated the associations between Mediterranean lifestyle-related dietary dimensions and daily sitting time. Two sets of models examined whether life satisfaction mediated the associations between MEDLIFE dietary consumption patterns and MEDLIFE dietary habits with sitting time. A second set of models tested whether social participation mediated the associations between these two MEDLIFE dietary dimensions and sitting time. Finally, an additional model examined whether life satisfaction mediated the association between social participation and sitting time. All mediation models were adjusted for age, sex, country or region, education level, employment status, BMI, smoking status, alcohol consumption, and self-reported health status. Indirect effects were estimated using a bootstrapping procedure with 5000 resamples and 95% confidence intervals. An indirect effect was considered statistically significant when the corresponding confidence interval did not include zero [47,48]. Prior to model interpretation, standard regression assumptions, including linearity, normality of residuals, homoscedasticity, and multicollinearity, were examined. Multicollinearity was assessed using variance inflation factors (VIF) and tolerance statistics, and the corresponding diagnostics for the fully adjusted model are presented in Supplementary Table S1. Because several conceptually related sleep indicators were entered simultaneously in the fully adjusted model, these diagnostics were considered particularly relevant when interpreting individual coefficients. All statistical tests were two-tailed, and statistical significance was set at *p* < 0.05.

## 3. Results

### 3.1. Descriptive Characteristics of the Study Population

The analytical sample comprised 4010 adults from ten Mediterranean and neighbouring countries. A detailed description of participant characteristics has been previously reported [10,35,36,38] and is summarized briefly here for context. The sample included participants of both sexes (40.5% male and 59.5% females) and diverse educational (58.8% with bachelor/master/doctorate degrees), occupational, (50.7% employed) and residential backgrounds (66.3% from urban environments), with wide variability in Mediterranean lifestyle adherence (31.1%, 46.8, and 22.1% for low, medium, and high adherence, respectively) and movement behaviours (60.3, 20.2, 19.5% for low, medium, and high PA categories and sitting time at 5.59 ± 3.04).

### 3.2. Hierarchical Regression Analyses Examining Correlates of Physical Activity

Table 1 reports the results of the hierarchical multiple linear regression analyses examining physical activity behaviours (log-transformed MET-min/week). Across the hierarchical models, the modest explained variance increased from 1.7% in M1 to 7.7% in M6, with the largest incremental improvement observed after the inclusion of psychological variables (ΔR^2^ = 0.018) and health-related variables (ΔR^2^ = 0.016), followed by the addition of social participation ancd technology-use indicators (ΔR^2^ = 0.010), sleep indicators (ΔR^2^ = 0.009) and Mediterranean lifestyle adherence (ΔR^2^ = 0.007). In the final model (M7), the included variables explained 7.2% of the variance in physical activity with a modest R^2^ = 0.072 (adjusted R^2^ = 0.069), indicating that a substantial proportion of variability in physical activity is likely explained by unmeasured factors. Higher physical activity outcomes were positively associated after adjustment with living in rural environment (B = 0.127, 95% CI: 0.064 to 0.190, *p* < 0.001), more favourable smoking status (B = 0.138, 95% CI: 0.078 to 0.197, *p* = 0.002) and higher alcohol use (B = 0.243, 95% CI: 0.161 to 0.325, *p* < 0.001), higher adherence to MEDLIFE dietary habits (B = 0.074, 95% CI: 0.043 to 0.105, *p* < 0.001), higher sleep latency (B = 0.004, 95% CI: 0.003 to 0.006, *p* = 0.020) and stress (B = 0.036, 95% CI: 0.020 to 0.051, *p* < 0.001), and higher social participation (B = 0.016, 95% CI: 0.011 to 0.021, *p* < 0.001). Conversely, lower physical activity outcomes was associated after adjustment with female sex (B = −0.296, 95% CI: −0.394 to −0.197, *p* < 0.001), higher BMI (B = −0.016, 95% CI: −0.026 to −0.006, β = −0.051, *p* < 0.001), higher sleep duration (B = −0.033, 95% CI: −0.061 to −0.005, *p* < 0.001), and higher anxiety (B = −0.072, 95% CI: −0.087 to −0.056, *p* < 0.001). Among these potential predictors, DASS anxiety showed the strongest standardized association (β = −0.226), although the magnitude of the association remained moderate. Variables such as age, region, education, employment status, marital status, insomnia, and technology use did not show significant association with IPAQ score in the final model. Collinearity diagnostics for the fully adjusted model are presented in Supplementary Table S1 (for physical activity) and Table S2 (for sedentary behaviour). Although most predictors were within acceptable ranges, sleep latency and efficiency showed elevated collinearity, and these coefficients should therefore be interpreted cautiously.

### 3.3. Hierarchical Regression Analyses Examining Correlates of Sedentary Behaviour

Table 2 reports the results of the hierarchical multiple linear regression analyses examining sedentary behaviour (daily sitting time). Across the successive models, the proportion of explained variance increased modestly from 0.6% in M1 to 3.1% in M6, with the largest incremental improvement observed after the inclusion of health-related variables and psychological outcomes (ΔR^2^ = 0.008), followed by the addition of Mediterranean lifestyle adherence (ΔR^2^ = 0.005), and finally the addition of sleep-related outcomes (ΔR^2^ = 0.003), social participation, and technology-use indicators (ΔR^2^ = 0.002).

In the final model (M7), the retained predictors accounted for a very small proportion of the variance (R^2^ = 0.024; adjusted R^2^ = 0.022), highlighting the limited explanatory power of the model Higher daily sitting time was positively associated after adjustment with higher education (B = 0.215, 95% CI: 0.142 to 0.289, *p* = 0.000), BMI (B = 0.026, 95% CI: 0.006 to 0.046, *p* = 0.010) and more favourable smoking status (B = 0.139, 95% CI: 0.022 to 0.256, *p* = 0.020) behaviours. Conversely, lower daily sitting time was associated after adjustment with better health status (B = −0.199, 95% CI: −0.363 to −0.036, *p* = 0.017), higher adherence to MEDLIFE dietary consumption patterns (B = −0.081, 95% CI: −0.129 to −0.033, *p* = 0.001), higher social participation (B = −0.013, 95% CI: −0.023 to −0.004, *p* = 0.007), and higher life satisfaction (B = −0.047, 95% CI: −0.069 to −0.025, *p* < 0.001). Among these potential predictors, education level showed the strongest standardized association (β = 0.09), followed by life satisfaction (β = −0.07), the adherence to the MEDLIFE dietary consumption (β = −0.052) and social participation, BMI, health status and smoking (β = |0.04|). Variables such as age, sex, region, employment and marital status, living environment, alcohol consumption, sleep pattern and DASS scores were not retained in the final model, except anxiety, which was retained but did not show significant association.

### 3.4. Exploratory Mediation Analyses Examining Indirect Associations Between Lifestyle Factors and Movement Behaviours

**Figure 1:**
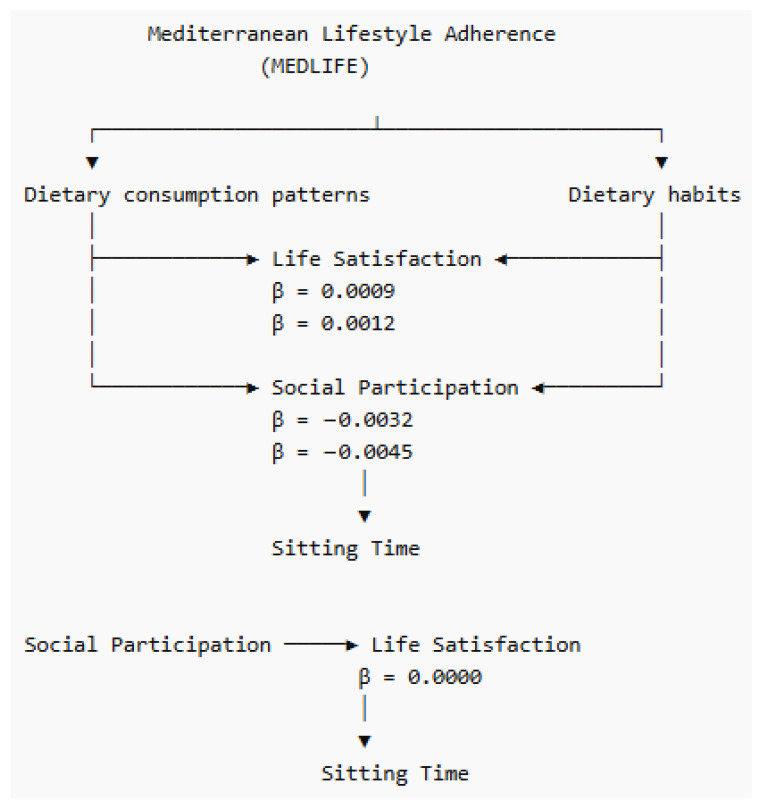
Conceptual mediation pathways linking Mediterranean lifestyle adherence and sedentary outcomes (Indirect β).

Exploratory bootstrapped mediation analyses are presented in Table 3 and Figure 1. Given that regression models indicated clearer associations between Mediterranean dietary dimensions, social participation, life satisfaction, and sitting time than for physical activity, mediation analyses focused on sedentary behaviour. Within the Mediterranean lifestyle–social participation pathway (models B1 and B2), social participation significantly mediated the associations between Mediterranean lifestyle dietary dimensions and sedentary behaviour (sitting time). Specifically, the indirect effect of MEDLIFE dietary consumption patterns on sitting time through social participation was small but statistically significant (indirect β = −0.0032, 95% CI: −0.0070 to −0.0002). Similarly, MEDLIFE dietary habits showed a significant indirect association with sitting time through social participation (indirect β = −0.0045, 95% CI: −0.0098 to −0.0004). Comparing the two pathways, the indirect effect was slightly larger for MEDLIFE dietary habits than for dietary consumption patterns, although both effects were small in magnitude and should be interpreted with caution. By contrast, the life satisfaction pathway was not supported (model A1 and A2). The indirect effects for both MEDLIFE dietary consumption patterns → life satisfaction → sitting time and MEDLIFE dietary habits → life satisfaction → sitting time were not statistically significant, as the bootstrap confidence intervals included zero. Likewise, life satisfaction did not mediate the association between social participation and sitting time, indicating no evidence of indirect effects through this pathway.

## 4. Discussion

The present study examined physical activity and sedentary behaviour as key movement-related outcomes within the MEDIET4ALL framework and showed that these two behaviours, although related, were associated with distinct but partially overlapping sets of sociodemographic, behavioural, and psychosocial correlates. Overall, physical activity was more strongly linked to behavioural activation-related and psychosocial variables, including Mediterranean dietary habits, sleep timing indicators, anxiety, stress, and social participation, whereas sedentary behaviour was more strongly associated with educational level, health status, Mediterranean dietary consumption patterns, life satisfaction, and social participation. The mediation analyses further indicated that social participation, rather than life satisfaction, may represent a modest behavioural pathway linking Mediterranean dietary dimensions with daily sitting time. Taken together, these findings reinforce the view that physical activity and sedentary behaviour should not be treated as simple opposites, but rather as distinct lifestyle domains embedded within broader Mediterranean lifestyle patterns and social contexts. However, given the cross-sectional design of the study, all associations and indirect effects should be interpreted cautiously and cannot be considered causal.

### 4.1. Distinct but Interconnected Determinants of Physical Activity and Sedentary Behaviour

A central finding of the present study is that physical activity and sedentary behaviour were not explained by the same set of correlates, despite both being movement-related outcomes. In the final adjusted models, physical activity was positively associated with rural living, lower smoking burden, alcohol use, Mediterranean dietary habits, longer sleep latency, stress, and social participation and negatively associated with female sex, higher body mass index, longer sleep duration, and anxiety. Sedentary behaviour, in contrast, was positively associated with higher educational level, higher body mass index, and more favourable smoking behaviour, whereas lower sitting time was associated with better self-reported health status, stronger adherence to Mediterranean dietary consumption patterns, higher social participation, and greater life satisfaction. These distinct patterns support the increasingly established idea that physical activity and sedentary behaviour are related but non-equivalent movement behaviours, each shaped by partly different determinants and each requiring specific analytical and intervention approaches [8,27].

This interpretation is strongly consistent with previous MEDIET4ALL findings. Earlier analyses from the MEDIET4ALL dataset showed marked cross-country variability in both physical activity and sitting time, and importantly, the two behaviours did not follow the same cross-country gradient. For example, some contexts displayed comparatively favourable physical activity profiles while also reporting high sitting time, reinforcing the notion that active living and sedentary accumulation may coexist within the same population [39,41].

The present findings extend this insight from cross-country descriptive comparisons to multivariable models and suggest that the distinction between these two domains is not only descriptive but also etiologically meaningful. Accordingly, interventions aimed at improving Mediterranean lifestyle behaviours should not presume that increasing physical activity will necessarily reduce sedentary time, or vice versa. In addition, broader contextual factors such as the built environment, occupational demands, and sociocultural norms, although not directly assessed in the present study, may further contribute to shaping movement behaviours and should be considered when interpreting these findings. This interpretation is supported by recent evidence showing that environmental characteristics (e.g., walkability and urban design), occupational context, and social environments are important determinants of both physical activity and sedentary behaviour across populations [49,50,51,52].

The physical activity findings are broadly in line with the wider epidemiological literature. The inverse association between female sex and physical activity, together with the positive association of rural living, is consistent with long-standing evidence showing that movement behaviour is strongly shaped by environmental opportunities, occupational patterns, gendered roles, and access to recreational or transport-related activity [26]. Likewise, the negative association between BMI and physical activity is coherent with the bidirectional relationship between excess body weight and reduced movement, although the present design does not allow conclusions regarding directionality. The positive association between stress and physical activity, alongside the negative association with anxiety, may appear counterintuitive at first glance, but it may reflect the fact that some individuals engage in physical activity as a coping strategy for stress, whereas anxiety may be more closely tied to behavioural inhibition or avoidance [53]. This interpretation is supported by evidence indicating differential behavioural responses to stress and anxiety, where stress may promote coping-related activity, whereas anxiety may inhibit behavioural activation [54,55,56]. The positive association between physical activity and alcohol consumption should be interpreted cautiously, as it may reflect residual confounding, social-patterning effects, or clustering of leisure-related behaviours rather than a health-promoting behavioural profile. Previous research has shown that physically active individuals may also report higher alcohol consumption, particularly in social and recreational contexts [57,58,59].

Sedentary behaviour, on the other hand, appeared to be more strongly embedded in educational, health, dietary, and psychosocial contexts. The positive association between educational level and sitting time is plausible and likely reflects occupational and academic demands, given that highly educated adults are more likely to be employed in white-collar and professional occupations characterized by desk-based and predominantly cognitively demanding sedentary tasks, which may contribute to higher levels of occupational sitting [60,61]. The positive association between sedentary behaviour and more favourable smoking status may appear unexpected. One possible explanation is that individuals with higher educational attainment, who are more likely to accumulate sedentary time through desk-based or office-based work, also tend to exhibit lower smoking prevalence, reflecting established socioeconomic gradients in health behaviours [62,63]. However, given the small effect size and low explained variance, this interpretation remains speculative. Similarly, the association of better self-reported health with lower sitting time aligns with earlier MEDIET4ALL analyses showing that sedentary behaviour was more consistently associated with poorer health status than physical activity behaviours were associated with better health status [10]. This distinction is also compatible with the broader sedentary behaviour literature, which has repeatedly shown that prolonged sitting is independently associated with adverse health outcomes, even among individuals who meet recommended physical activity levels [6,7,8,9]. Together, these findings suggest that sedentary behaviour may carry its own health and behavioural correlates rather than simply representing the absence of physical activity.

Another important contribution of the present study is the differential role of Mediterranean lifestyle dietary dimensions. By analyzing the two dietary MEDLIFE domains separately, this paper shows that Mediterranean dietary habits were positively associated with physical activity, whereas Mediterranean dietary consumption patterns were inversely associated with sedentary behaviour. This distinction is informative because it suggests that different aspects of Mediterranean lifestyle adherence may relate to different movement outcomes. Mediterranean dietary consumption patterns primarily reflect the quality and composition of food intake, including higher consumption of fruits, vegetables, legumes, fish, and olive oil and lower consumption of processed foods, thereby capturing adherence to the core nutritional profile of the Mediterranean diet [45]. In contrast, Mediterranean dietary habits reflect behavioural aspects of eating, including moderation, regulation, and restraint (e.g., limiting salt, sugar, snacking, and nibbling), which may indicate broader self-regulatory capacities and healthier behavioural routines. These characteristics may reflect underlying self-regulatory processes that have been linked to higher engagement in health-promoting behaviours, including physical activity. These behavioural characteristics may align more closely with active lifestyles, whereas overall dietary composition may be more strongly embedded within broader lifestyle profiles associated with lower sedentary accumulation. This extends previous MEDIET4ALL work demonstrating that Mediterranean lifestyle adherence is shaped by a multidimensional combination of demographic, behavioural, and psychosocial determinants rather than by dietary intake alone [19].

Beyond these direct associations, the present study also explored potential indirect pathways linking Mediterranean lifestyle dimensions with sedentary behaviour.

### 4.2. Mediterranean Lifestyle, Social Participation, and Indirect Pathways to Sedentary Behaviour

The current results also suggest social participation as a potential psychosocial factor. Social participation was positively associated with physical activity and negatively associated with sitting time, and the mediation analyses indicated small-magnitude indirect associations between both Mediterranean dietary dimensions and sedentary behaviour through social participation. The present mediation findings therefore suggest that Mediterranean dietary dimensions may relate to lower sitting time partly through social participation, although these effects were small in magnitude and should be interpreted with caution, potentially reflecting a small statistical pathway with limited practical relevance. In contrast, life satisfaction did not mediate these links. This pattern may suggest that social environment could play a more prominent role than subjective well-being in linking Mediterranean lifestyle dietary behaviours with sitting time. This is plausible within the MEDIET4ALL framework, given that previous analyses have repeatedly identified social participation as one of the most variable and context-sensitive lifestyle dimensions across countries [39,40,41]. It is also consistent with broader evidence indicating that social participation and social capital are positively related to physical activity and may help reduce inactivity by creating opportunities, norms, and support structures for movement [34]. The present mediation findings therefore provide a small statistical signal suggesting that Mediterranean dietary dimensions may relate to lower sitting time partly through social engagement, although these effects should be interpreted with caution given their limited magnitude.

By contrast, the life satisfaction pathways were not supported in the sedentary-behaviour mediation analyses, even though life satisfaction was independently associated with lower sitting time in the final regression model. This may suggest that, in the context of movement behaviours, life satisfaction is more likely to operate as a parallel correlate rather than a meaningful mediator of dietary and social influences. In other words, adults with more favourable dietary patterns and stronger social participation may indeed report both lower sitting time and better life satisfaction, but the present results do not support the idea that life satisfaction is the key mechanism linking these behaviours. This interpretation is broadly consistent with the recent systematic review by Godos ey al. [64], which showed that adherence to the Mediterranean diet was generally associated with better health-related quality of life, with the most consistent findings observed for physical domains rather than uniformly across all psychosocial dimensions. In that light, the present findings may suggest that, although healthier dietary patterns and psychosocial resources can co-occur with greater life satisfaction, their relationship with sedentary behaviour may be more directly associated with behavioural and social pathways rather than operating through subjective well-being. This is interesting in light of another MEDIET4ALL paper [65], where life satisfaction played a more prominent mediating role in the psychological domain. The contrast between the two papers may therefore suggest that sleep and life satisfaction are more central for psychological outcomes, whereas social participation may be more central for sedentary behaviour. This interpretation also aligns with the broader conclusion of Godos et al. [64] that Mediterranean diet adherence appears to support overall quality of life but that the strength and consistency of associations may differ across domains and populations. That difference adds conceptual value to the MEDIET4ALL programme by showing that different lifestyle outcomes may be organized around different core pathways. However, given the small magnitude of the observed indirect effects, the mediation findings should be interpreted as exploratory rather than indicative of strong behavioural mechanisms.

From a public health perspective, these findings support a more integrated yet differentiated approach to movement promotion. Current WHO guidance already emphasizes that adults should both increase physical activity and reduce sedentary time, recognizing these as related but distinct health targets [27]. The present results reinforce that position and suggest that Mediterranean lifestyle promotion may benefit from addressing not only movement behaviours themselves but also the broader social and behavioural environments in which they occur [19,66]. For physical activity, this may involve context-sensitive strategies that account for sex differences, rural–urban opportunities, social participation, and psychological states. For sedentary behaviour, strategies may need to focus more explicitly on occupational sitting, educational contexts, and opportunities for social engagement that interrupt prolonged sitting. Previous MEDIET4ALL work already suggested that context-sensitive, culturally adapted, and socially embedded interventions are likely to be more effective than one-size-fits-all approaches, and the present study strengthens that conclusion specifically for movement outcomes.

### 4.3. Strengths and Limitations

This study has several strengths. It is based on a large multinational sample drawn from ten Mediterranean and neighbouring countries and uses a standardized multilingual e-survey framework with excellent cross-language reliability. It is also, to our knowledge, the first MEDIET4ALL paper to examine physical activity and sedentary behaviour as primary outcomes within the same analytical framework. A further strength is the decision to analyze the two dietary MEDLIFE domains separately, which made it possible to detect distinct associations for physical activity and sitting time that would likely have been obscured by use of a total score. Finally, the exploratory mediation analyses added conceptual depth by testing whether psychosocial variables could help explain the observed associations between Mediterranean dietary dimensions and sedentary behaviour.

Several limitations should also be acknowledged. First, the cross-sectional design precludes any inference about temporal direction or causality. Second, physical activity and sitting time were assessed by self-report, which may introduce recall bias, social desirability bias, and systematic measurement error, including overestimation of physical activity and limited precision in capturing context-specific sedentary behaviours, particularly for sitting time assessed using a single-item measure. Third, the explained variance of the final models was modest for physical activity and particularly low for sedentary behaviour, indicating limited explanatory power and suggesting that key determinants of movement behaviours were not captured. In addition, the simultaneous inclusion of several related sleep indicators in the fully adjusted physical activity model may have introduced collinearity, which should be considered when interpreting individual regression coefficients. Relatedly, important environmental and occupational determinants were not directly assessed; factors such as the built environment, occupational demands, and sociocultural context may account for additional variability in both physical activity and sitting time. Fourth, some predictors were coded categorically or ordinally, which limits fine-grained interpretation of dose–response patterns. In addition, although the large and multi-country sample enhances external validity, potential sampling bias related to the online, volunteer-based recruitment approach (e.g., self-selection and differential digital access to the survey) cannot be excluded, which may limit the generalizability of the findings. Finally, the mediation models were exploratory and the indirect effects, although statistically informative, were small in magnitude. Accordingly, these pathways should be understood as preliminary statistical signals rather than established mechanisms. Future studies should use longitudinal or intervention designs, together with objective measures of physical activity and sedentary time, to test whether the relationships observed here are reproducible and temporally robust.

### 4.4. Implications and Future Directions

The present findings have several implications for both research and public health practice. First, the clear differentiation observed between physical activity and sedentary behaviour reinforces the need to conceptualize movement behaviours as distinct, although interrelated, lifestyle domains. While Mediterranean dietary habits were more closely associated with physical activity, dietary consumption patterns were more strongly linked to sedentary behaviour, suggesting that different components of the Mediterranean lifestyle may influence movement behaviours through partially independent pathways. This distinction has practical relevance for intervention design, indicating that strategies aimed at increasing physical activity may require different behavioural targets than those aimed at reducing sedentary time. From an applied perspective, these findings have implications across multiple sectors, including public health policy, workplace health promotion, education, and community-based programmes. Second, the consistent association between social participation and both movement behaviours, together with its mediating role in the relationship between Mediterranean dietary dimensions and sedentary behaviour, highlights the importance of social and environmental contexts. These findings suggest that interventions promoting social engagement and community participation may contribute not only to improved well-being but also to more favourable movement behaviour profiles. For example, workplace interventions may focus on reducing prolonged sitting through environmental modifications and active breaks, whereas community and educational settings may prioritize enhancing social participation and access to structured or informal physical activity opportunities. From a public health and policy perspective, the results support integrated lifestyle approaches that combine dietary, social, and behavioural components rather than addressing physical activity or sedentary behaviour in isolation. In particular, reducing sedentary time may require specific attention even among individuals who meet physical activity recommendations, as these behaviours appear to be influenced by distinct determinants.

Future research should prioritize longitudinal and experimental designs to clarify temporal relationships and potential causal pathways. The use of objective measures (e.g., accelerometry) would further strengthen the assessment of movement behaviours. In addition, future studies should incorporate broader environmental and contextual determinants, such as occupational demands, urban design, and sociocultural influences, which may play a key role in shaping both physical activity and sedentary behaviour across populations.

## 5. Conclusions

In conclusion, the present study indicates that physical activity and sedentary behaviour are associated with distinct but partially overlapping sets of lifestyle, psychosocial, health-related, and sociodemographic correlates within the MEDIET4ALL framework. Mediterranean dietary habits were more closely linked to physical activity, whereas Mediterranean dietary consumption patterns were more strongly associated with sedentary behaviour. Social participation emerged as an important correlate of both movement behaviours and, in the mediation analyses, as a small-magnitude pathway linking Mediterranean dietary dimensions with lower sitting time. Taken together with the implications outlined above, these findings support the need for integrated, multidimensional strategies that address physical activity and sedentary behaviour as complementary but distinct public health targets. From a practical and policy perspective, interventions should concurrently promote increases in physical activity and reductions in sedentary time through context-specific strategies, particularly in occupational and educational settings where sitting time is high. Policies that integrate movement-promoting environments with social engagement opportunities (e.g., workplace active breaks, community-based programmes) may be especially relevant for supporting sustainable behaviour change.

## Figures and Tables

**Table 1:** Hierarchical multiple linear regression analyses examining associations between sociodemographic, health-related, Mediterranean dietary behaviours, sleep, psychological, and psychosocial correlates of physical activity.

Predictor	M1 β	M2 β	M3 β	M4 β	M5 β	M6 β	M7 B (95% CI)	M7 β	M7 *p*
Age	0.031	0.052	0.032	0.019	0.006	0.032			
Sex	−0.101	−0.099	−0.103	−0.104	−0.104	−0.098	−0.296 (−0.394; −0.197)	−0.097	0.000
Region	0.008	0.002	−0.004	−0.015	−0.010	−0.008			
Education	0.047	0.041	0.035	0.034	0.025	0.015			
Employment	0.040	0.027	0.031	0.033	0.038	0.031			
Marital status	−0.027	−0.025	−0.025	−0.024	−0.025	−0.025			
Living environment	0.059	0.052	0.055	0.053	0.055	0.061	0.127 (0.064; 0.19)	0.064	0.000
BMI		−0.066	−0.062	−0.061	−0.060	−0.057	−0.016 (−0.026; −0.006)	−0.051	0.000
Smoking (higher = less smoking)		0.074	0.071	0.068	0.068	0.067	0.138 (0.078; 0.197)	0.074	0.002
Alcohol		0.094	0.093	0.094	0.112	0.099	0.243 (0.161; 0.325)	0.098	0.000
Health status		−0.008	−0.013	−0.010	−0.024	−0.026			
MEDLIFE dietary consumption patterns			0.032	0.035	0.036	0.028			
MEDLIFE dietary habits			0.076	0.080	0.077	0.069	0.074 (0.043; 0.105)	0.076	0.000
Sleep duration				−0.076	−0.063	−0.069	−0.033 (−0.061; −0.005)	−0.038	0.000
Sleep latency				0.187	0.205	0.211	0.004 (0.003; 0.006)	0.096	0.020
Sleep efficiency				0.121	0.120	0.127			
Subjective sleep quality				−0.038	−0.018	−0.029			
ISI total score				−0.062	−0.013	−0.023			
SLSQ total score					0.012	−0.008			
DASS depression					−0.036	−0.018			
DASS anxiety					−0.195	−0.216	−0.072 (−0.087; −0.056)	−0.226	0.000
DASS stress					0.127	0.123	0.036 (0.02; 0.051)	0.112	0.000
SSPQ total score						0.113	0.016 (0.011; 0.021)	0.106	0.000
STuQL total score						0.003			
No. of predictors	7	11	13	18	22	24	11		
R^2^	0.017	0.034	0.041	0.049	0.067	0.077	0.072		
Adjusted R^2^	0.015	0.031	0.037	0.045	0.062	0.071	0.069		
ΔR^2^	0.017	0.016	0.007	0.009	0.018	0.01	0.072		
F change	9.28	15.38	13.82	6.69	17.42	20.01	25.99		

**Table 2:** Hierarchical multiple linear regression analyses examining sociodemographic, health-related, Mediterranean dietary behaviour, sleep, psychological, and psychosocial correlates of sedentary behaviour.

Predictor	M1 β	M2 β	M3 β	M4 β	M5 β	M6 β	M7 B (95% CI)	M7 β	M7 *p*
Age	0.019	−0.006	0.008	0.014	0.012	0			
Sex	−0.02	−0.017	−0.011	−0.017	−0.02	−0.023			
Region	−0.023	−0.011	−0.005	−0.008	−0.013	−0.014			
Education	0.071	0.075	0.079	0.081	0.084	0.088	0.215 (0.142; 0.289)	0.093	0.000
Employment	0.004	−0.002	−0.005	−0.009	−0.005	−0.002			
Marital status	−0.007	−0.015	−0.016	−0.017	−0.01	−0.01			
Living environment	−0.013	−0.017	−0.021	−0.023	−0.023	−0.026			
BMI		0.053	0.05	0.049	0.045	0.044	0.026 (0.006; 0.046)	0.042	0.010
Smoking (higher = less smoking)		0.035	0.038	0.036	0.038	0.039	0.139 (0.022; 0.256)	0.037	0.020
Alcohol		0.012	0.013	0.008	0.009	0.015			
Health status		−0.059	−0.055	−0.047	−0.042	−0.041	−0.199 (−0.363; −0.036)	−0.039	0.017
MEDLIFE dietary consumption patterns			−0.051	−0.048	−0.045	−0.042	−0.081 (−0.129; −0.033)	−0.052	0.001
MEDLIFE dietary habits			−0.039	−0.035	−0.029	−0.025			
Sleep duration				0.027	0.03	0.033			
Sleep latency				−0.005	−0.008	−0.011			
Sleep efficiency				−0.042	−0.045	−0.048			
Subjective sleep quality				−0.034	−0.024	−0.019			
ISI total score				0.005	−0.013	−0.009			
SLSQ total score					−0.052	−0.044	−0.047 (−0.069; −0.025)	−0.07	0.000
DASS depression					0.059	0.052			
DASS anxiety					−0.088	−0.08	0.004 (−0.017; 0.025)	0.007	0.690
DASS stress					0.051	0.052			
SSPQ total score						−0.048	−0.013 (−0.023; −0.004)	−0.044	0.007
STuQL total score						−0.015			
N	7	11	13	18	22	24	8		
R^2^	0.006	0.014	0.018	0.022	0.029	0.031	0.024		
Adjusted R^2^	0.004	0.011	0.015	0.017	0.024	0.026	0.022		
ΔR^2^	0.006	0.008	0.005	0.003	0.008	0.002	0.024		
F change	3.437	7.766	9.547	2.715	7.864	4.242	12.14		

**Table 3:** Bootstrapped mediation analyses examining indirect associations between lifestyle factors and movement behaviours in the MEDIET4ALL sample.

Pathway	Direct β	Indirect β	95% CI
A1: MEDLIFE dietary consumption patterns → SLSQ → sitting time	0.0316	0.0009	−0.0009 to 0.0030
A2: MEDLIFE dietary habits → SLSQ → sitting time	0.0778	0.0012	−0.0032 to 0.0056
B1: MEDLIFE dietary consumption patterns → SSPQ → Sitting time	−0.0860	−0.0032	−0.0070 to −0.0002
B2: MEDLIFE dietary habits → SSPQ → Sitting time	−0.0898	−0.0045	−0.0098 to −0.0004
C: Social participation → SLSQ → sitting time	0.0143	0.0000	−0.0011 to 0.0012

## Data Availability

The datasets generated and analyzed during the current study are not publicly available at this time as further analyses are ongoing, and additional publications based on these data are in preparation. Data may be made available upon reasonable request to the corresponding author once all planned analyses and publications are completed.

## Supplementary Materials

The following supporting information can be downloaded at: https://www.mdpi.com/article/10.3390/sports14050186/s1, Table S1: Collinearity diagnostics for predictors included in the fully adjusted model (model 6) examining correlates of IPAQ; Table S2: Collinearity diagnostics for predictors included in the fully adjusted model (model 6) examining correlates of Sitting time.
