# Supplementary material for: Physical Activity and Sedentary Behaviour in the MEDIET4ALL Study: Associations with Mediterranean Lifestyle, Sleep, and Psychosocial Well-Being, with Mediation Analyses
Source: Sports (Basel). 2026 May 6;14(5):186. doi: 10.3390/sports14050186 (PMC13211037; doi:10.3390/sports14050186)
Supplement: Supplementary file 1 [file sports-14-00186-s001.zip › sports-4274652-supplementary.pdf]

Supplementary Table S1. Collinearity diagnostics for predictors included in the fully adjusted model (model 6) examining correlates of IPAQ

Predictor	Tolerance	VIF
Age	0.522	1.916
Sex	0.872	1.147
Region	0.905	1.105
Education	0.902	1.109
Employment	0.909	1.100
Marital status	0.648	1.544
Living environment	0.940	1.064
BMI	0.858	1.165
Smoking	0.926	1.080
Alcohol	0.874	1.144
Health status	0.824	1.214
MEDLIFE dietary consumption patterns	0.927	1.079
MEDLIFE dietary habits	0.887	1.127
Sleep duration	0.418	2.390
Sleep latency	0.065	15.499
Sleep efficiency	0.060	16.732
Subjective sleep quality	0.629	1.589
ISI total score	0.495	2.022
SLSQ total score	0.752	1.329
DASS depression	0.290	3.450
DASS anxiety	0.331	3.020
DASS stress	0.306	3.267
SSPQ total score	0.796	1.257
STuQL total score	0.989	1.011

VIF = variance inflation factor. Tolerance = 1/VIF. Diagnostics were derived from the fully adjusted physical activity model (Model 6). Elevated VIF values for some sleep-related variables likely reflect overlap among conceptually related sleep indicators entered simultaneously.

Supplementary Table S2. Collinearity diagnostics for predictors included in the fully adjusted model (model 6) examining correlates of Sitting time

Predictor	Tolerance	VIF
Age	0.523	1.913
Sex	0.870	1.150
Region	0.902	1.108
Education	0.902	1.109
Employment	0.911	1.097
Marital status	0.646	1.547
Living environment	0.943	1.061
BMI	0.858	1.166
Smoking	0.926	1.080
Alcohol	0.872	1.147
Health status	0.827	1.210
MEDLIFE dietary consumption patterns	0.926	1.080
MEDLIFE dietary habits	0.893	1.119
Sleep duration	0.413	2.421
Sleep latency	0.065	15.431
Sleep efficiency	0.060	16.686
Subjective sleep quality	0.621	1.611
ISI total score	0.486	2.056
SLSQ total score	0.748	1.337
DASS depression	0.284	3.515
DASS anxiety	0.332	3.008
DASS stress	0.299	3.342
SSPQ total score	0.794	1.260
STuQL total score	0.989	1.011

VIF = variance inflation factor. Tolerance = 1/VIF. Diagnostics were derived from the fully adjusted sedentary behaviour model (Model 6). Elevated VIF values for some sleep-related variables likely reflect overlap among conceptually related sleep indicators entered simultaneously.
